# 3D-Planned, Patient-Specific Distal Radius Reconstruction with a Vascularized Double-Barrel Free Fibular Graft After Secondary Aneurysmal Bone Cyst

**DOI:** 10.3390/jcm15051857

**Published:** 2026-02-28

**Authors:** Bita Kallenbach, Philipp Honigmann, Martin Haug, Marco Keller

**Affiliations:** 1 Hand and Peripheral Nerve Surgery, Department of Orthopaedic Surgery and Traumatology, Kantonsspital Baselland (Bruderholz, Liestal, Laufen), 4101 Bruderholz, Switzerland; 2 Medical Additive Manufacturing Research Group (MAM), Department of Biomedical Engineering, University of Basel, 4123 Allschwil, Switzerland; 3 Department of Biomedical Engineering and Physics, Amsterdam UMC, University of Amsterdam, Amsterdam Movement Sciences, 1105 Amsterdam, The Netherlands; 4 Department of Plastic, Reconstructive, Aesthetic, and Hand Surgery, University Hospital Basel, 4031 Basel, Switzerland; 5 Hand and Peripheral Nerve Surgery, Department of Orthopaedic Surgery, Traumatology and Hand Surgery, Spital Limmattal, 8952 Schlieren, Switzerland

**Keywords:** aneurysmal bone cyst, radius, radius fractures, pathologic fractures, reconstructive surgical procedures, vascularized fibula graft, 3D planning, computer-assisted surgery, secondary bone lesions, osteotomy, computed tomography

## Abstract

**Background/Objectives**: An Aneurysmal Bone Cyst (ABC) is a rare benign osteolytic bone lesion with locally destroying growth. It occurs mostly in the first two decades of life, rarely in older patients, and commonly affects the metaphysis. Clinical presentation includes pain and pathologic fractures. While most ABCs occur as primary lesions, there is an entity of secondary (reactive) ABC following osseous lesions such as fractures. We report a rare case of a secondary aneurysmal bone cyst of the distal radius following a distal radius fracture 4 years prior, with subsequent treatment and reconstruction. **Methods**: A 67-year-old female patient presented with a pathologic distal forearm fracture with radiologically expansive lytic bone lesion of the metaphysis of the distal radius, suspicious of an ABC. A biopsy and primary fracture management with an external fixator were performed due to the unclear dignity of the lesion. The diagnosis of an ABC was confirmed in the biopsy. The tumor resection and reconstruction were performed with a vascularized free fibula graft (ipsilateral, double barrel), using patient-specific 3D-printed osteotomy templates. **Results**: Follow-up radiographs showed excellent bone union with progressive remodeling. The functional outcome was very good with almost the same range of motion and grip strength as the contralateral side. No limitation in everyday life and no donor site morbidity was reported. **Conclusions**: ABC is a rare benign bone tumor the treatment of which consists of complete resection and reconstruction. Reconstruction of the distal radius can be achieved with a fibula graft. In our case, an excellent result was achieved with patient-specific osteotomy templates. Only a few cases of ABC in the distal radius and at this age have been reported; nevertheless, it should be considered as a differential diagnosis for osteolytic bone lesions

## 1. Introduction

Aneurysmal bone cysts (ABCs) are rare benign osteolytic bone lesions with expansile growth, occurring most commonly in the second decade of life with an incidence of 0.14 to 0.32 per 100’000 individuals [1,2,3,4,5,6,7]. They are typically found in the metaphysis of the long bones such as the distal femur, humerus, tibia, or ulna [6]; fewer than 5% are localized in the hand [2]. ABCs are considered to grow locally aggressively, without forming metastases [6].

ABCs can be divided into primary and secondary lesions, of which the majority are secondary [8]. The primary lesion appears as a true mesenchymal neoplasm, mostly exhibiting the USP6 and CDH11 gene abnormalities. Secondary ABCs may mimic morphology of the primary ABCs, without expressing the mentioned gene abnormalities [9]. They may result from primary lesions such as non-ossifying fibroma, chondroblastoma, solitary bone cyst, giant cell tumors, hemangioma, osteoblastoma, giant-cell reparative granuloma, fibrous dysplasia, and fibromyxoma [1,4]. Other authors have reported post-traumatic secondary ABCs [10,11,12]. Previous studies have shown evidence of an altered venous circulation resulting in an arteriovenous fistula, eventually leading to bone resorption [6,13,14]. The theory of a blood-flow alteration is supported by other authors in post-traumatic secondary ABCs [10,11,12,13]. However, the pathogenesis of the secondary ABC remains not fully understood.

There is still no consensus regarding treatment recommendations. Options include en bloc resection, intralesional curettage (with or without local adjuvants), embolization, sclerotherapy with polidocanol [15], radiotherapy, or medical treatment with bisphosphonates [16,17,18].

Computed tomography-based patient-specific surgical treatments have been introduced in other contexts, e.g., for the management of complex tibial plateau fractures [19]. In the distal radius, several case series demonstrated 3D-planned, patient-specific planning for corrective osteotomies [20,21]. Despite reports about 3D-planned, patient-specific templates for corrective osteotomies, there is a lack of documented cases about reconstruction of the distal radius after a tumor resection using this technique. We show a case of a 3D-planned, patient-specific distal radius reconstruction with a vascularized double-barrel free fibular graft after a secondary aneurysmal bone cyst.

## 2. Methods and Results

**Figure 1:**
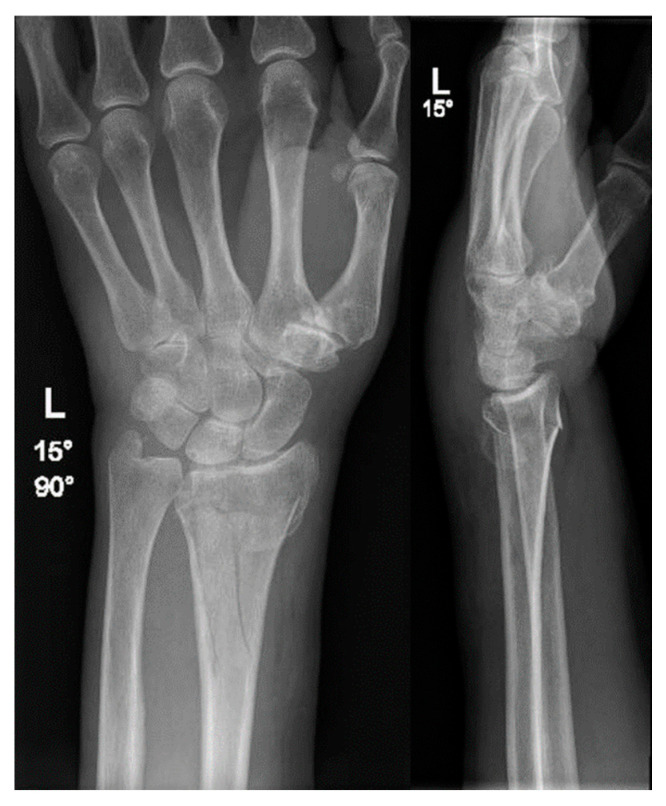
X-ray image in two planes of the initial fracture of the distal radius 4 years prior.

A 67-year-old female presented in our emergency room after she had fallen off of her bike while standing. The patient had a history of an intraarticular distal radius fracture (AO 23 C1) on the left arm 4 years prior (Figure 1) that had been treated by volar plate osteosynthesis. Otherwise, she was healthy. The osteosynthesis material had been removed.

**Figure 2:**
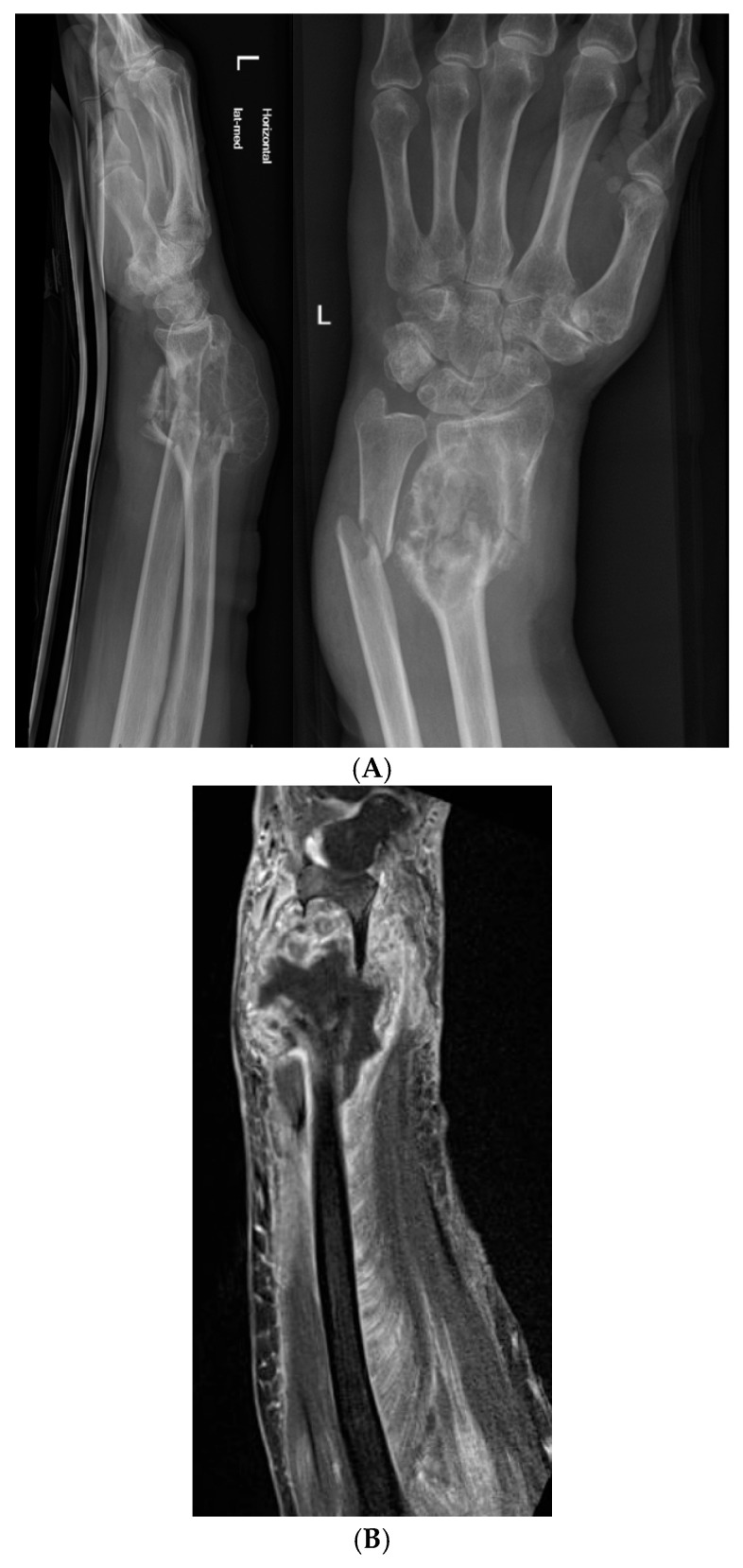
(**A**) X-ray in two planes of the perilesional fracture, (**B**) coronal T2 MRI scan.

The patient presented clinically with pain following her fall, as well as a palpable mass on her left wrist. A dislocated fracture of the ulna and a dislocated pathological fracture of the radius on the left forearm were detected using a conventional X-ray (Figure 2A) and CT. On current presentation, in the radial metaphysis, a lytic lesion with a sclerosed margin was found, suspicious of an ABC.

An MRI was performed for further diagnostics, which showed a solitary expansive lesion; however, there was no sign of fluid–fluid levels (Figure 2B).

**Figure 3:**
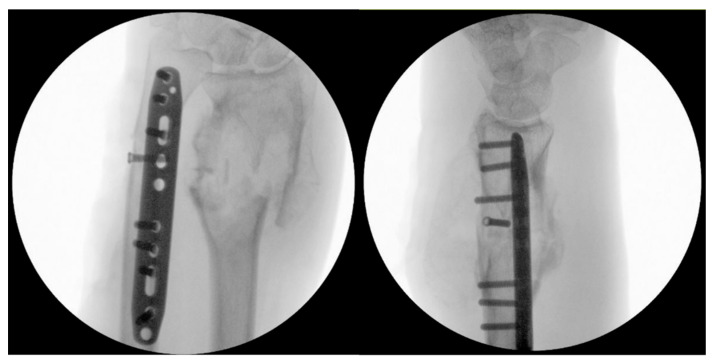
Intraoperative fluoroscopy showing osteosynthesis of the ulna.

The lesion was biopsied; furthermore, a fixation of the distal radius fracture using an external fixator and osteosynthesis of the ulnar fracture was performed (Figure 3).

Histopathological examination showed reactive bone formation with spindle-cell-proliferation and osteoclast-like giant cells. There were signs of diffuse hemorrhage, cystic formations, and septa. No signs of malignancy could be detected. The suspicion of an ABC was histologically confirmed, although the constellation was unusual with regard to the patient’s age.

**Figure 4:**
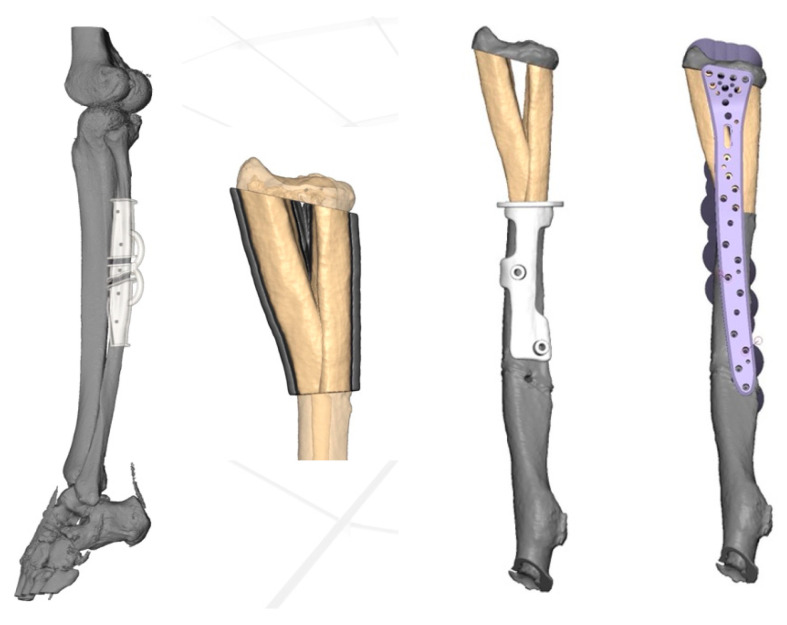
Patient-specific 3D planning of the osteotomy template.

In accordance with the regional tumor board, a treatment plan was developed. An en bloc resection of the lytic lesion was chosen. Reconstruction of the distal radius using an ipsilateral vascularized double-barrel fibula graft was selected. To reproduce the physiological shape of the distal radius metaphysis, the reconstruction was 3D planned and involved the use of patient-specific 3D-printed osteotomy templates (Figure 4). The computed tomography images of the affected forearm and the lower leg planned as the harvesting site were performed using a SIEMENS Biograph 128 scanner (Siemens AG, Munich, Germany) with an in-plane resolution of 0.25 mm × 0.25 mm and a slice thickness of 0.37 mm. Bone segmentation (radius and fibula) and osteotomy planning was conducted using Materialise Mimics 23.0 software (Materialise NV, Leuven, Belgium). A virtual osteotomy was performed by a medical engineer in order to fill the tumor resection site with the V-shaped double barrel autograft press-fit and according osteotomy guides were designed. Eventually, the plate fixation of the radius and the interposed graft were planned with a standard Aptus XL volar locking plate 2.5 (Medartis AG, Basel, Switzerland). The consent of the patient was obtained.

**Figure 5:**
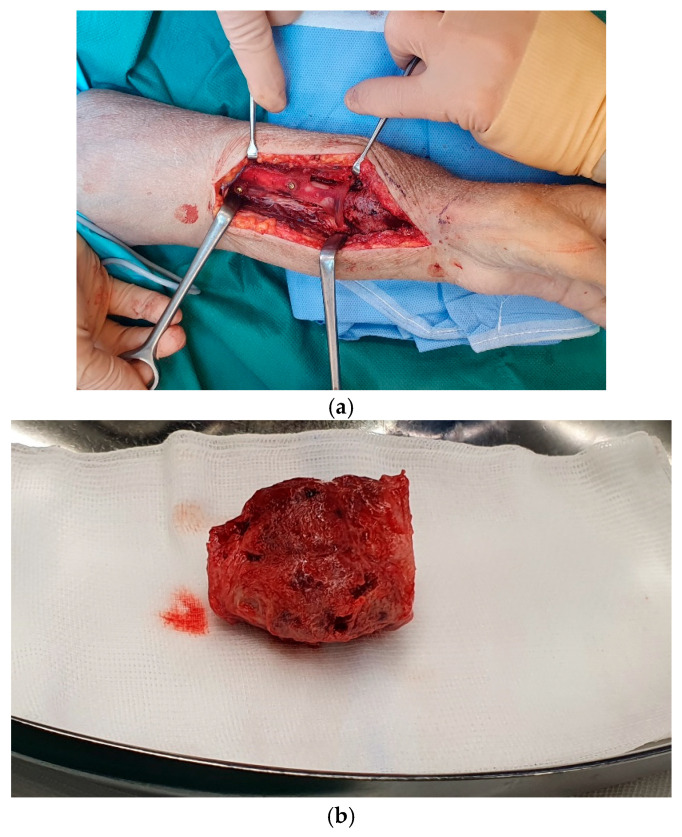
(**a**) Exposition of the lesion by a modified Henry approach, (**b**) excised lesion.

The operation was performed under general anesthesia. The skin was cut open in the axis of the fibula, subcutaneous preparation to the bone was performed, and a remainder of the soleus muscle was left on the fibula. An anterior approach to the vessels through the interosseus membrane was chosen. Distally, the fibular artery and vein were cut off, and the proximal and distal osteotomy was then performed with the templates. At the distal radius, a modified Henry approach was performed, exposing the tumor (Figure 5a).

The proximal osteotomy was carried out, using the patient-specific osteotomy template, thus excising the ABC (Figure 5b).

**Figure 6:**
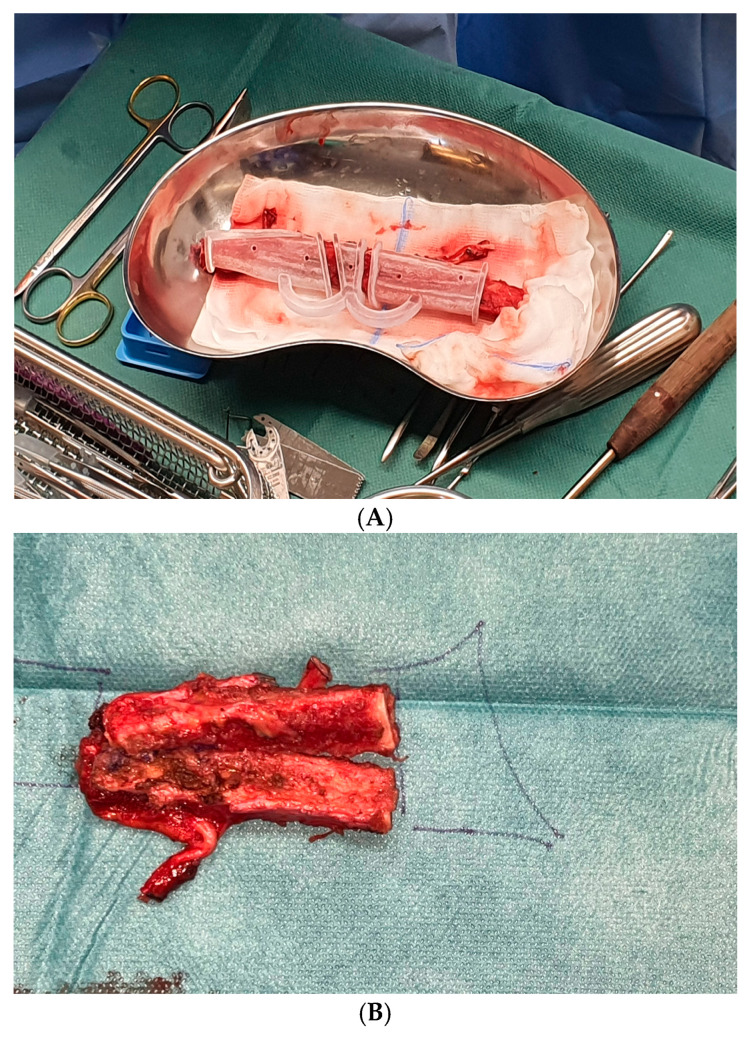
(**A**) Excision of the fibula using the 3D-planned osteotomy template, (**B**) fibula after free-hand osteotomy.

The initial plan was to apply the osteotomy guides in situ and resect only the required portions of the tibia. However, this had to be adjusted because placing the guides in situ risked compromising the vascular pedicle. Therefore, a slightly longer segment was excised. The pedicle could be protected more accurately on-table while applying the osteotomy guides and trimming the graft free-hand into the planned dimensions (Figure 6A,B).

**Figure 7:**
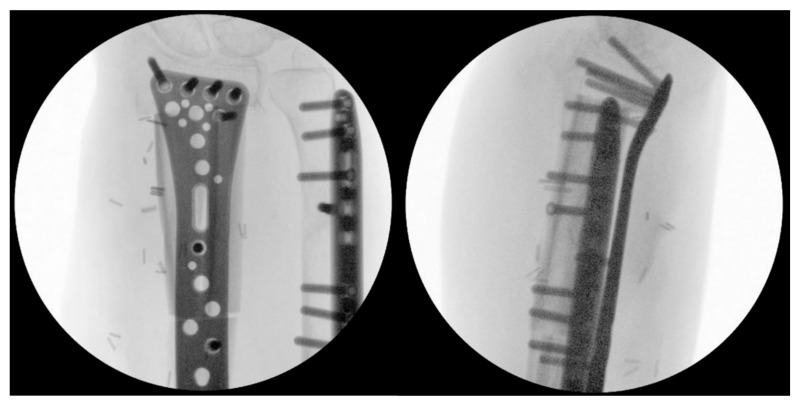
Intraoperative fluoroscopy showing the reconstructed radius in two planes with the physiological length and palmar tilt.

The graft was inserted and secured with an osteosynthesis plate. Intraoperative fluoroscopy showed the physiological length, palmar tilt, and shape of the distal radius (Figure 7).

## 3. Results

The postoperative course was uneventful and anticoagulation was achieved with unfractionated heparin during the first 24 h, after which the medication was adapted to acetylsalicylic acid for 6 weeks. Immobilization was carried out with a splint for 6 weeks with no mobilization of the wrist.

**Figure 8:**
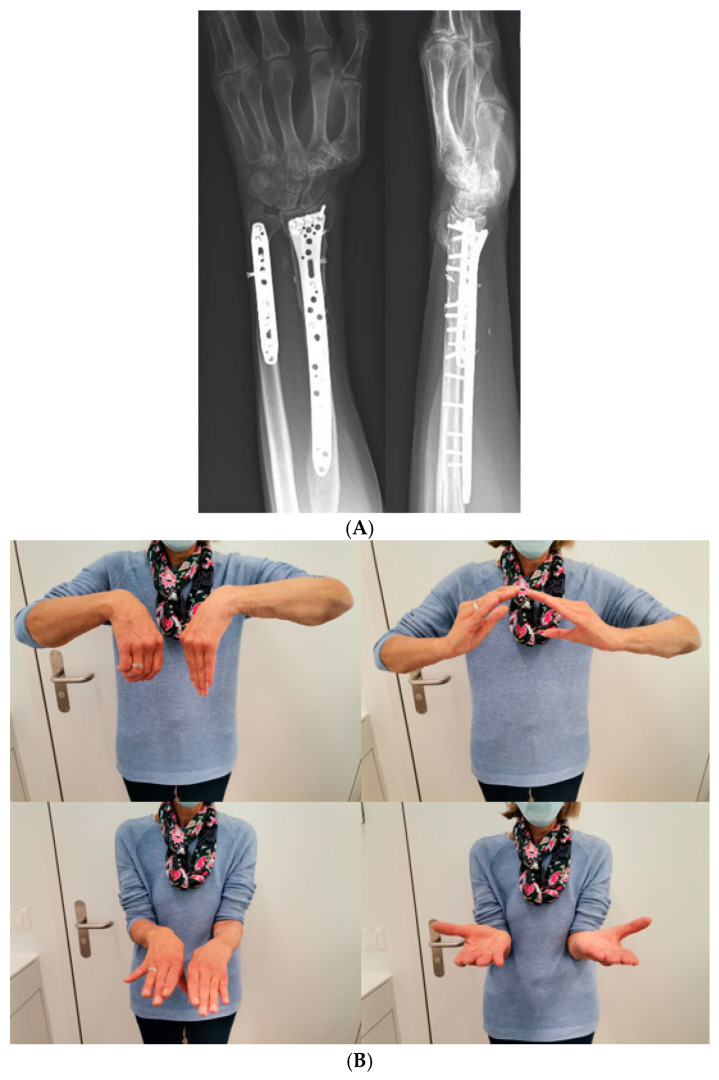
(**A**) X-ray in two planes at follow-up after 6 months, (**B**) clinical result after 6 months.

At follow-up after 6 months, the patient showed a good radiological and clinical result (Figure 8A,B).

**Figure 9:**
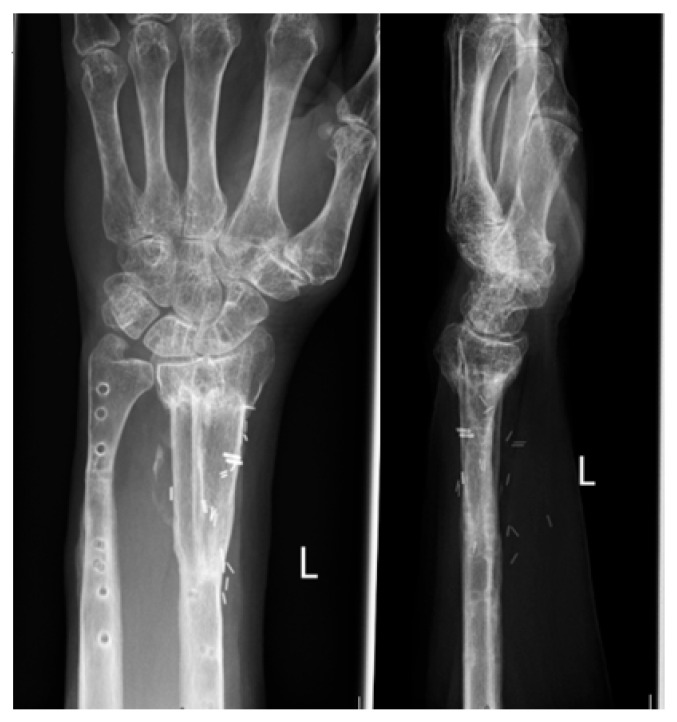
Follow-up at 6 weeks postoperatively after plate removal.

Regarding the distal radius, she had little to no pain. An active range of motion of the patient’s operated wrist is shown in Table 1. After 18 months, the postoperative reconstruction showed complete consolidation on X-ray with excellent range of motion. Thus, the removal of osteosynthesis material was performed at 20 months postoperatively (Figure 9).

There was minimal donor site morbidity, and the patient was able to walk with full weight bearing after a short time and even managed a 10 km hike.

## 4. Discussion

Aneurysmal bone cysts are rare benign osteolytic lesions. So far, there have been no reported post-traumatic secondary ABCs in the distal radius, especially among people in this age group.

Patients usually present with local pain and swelling [22], and occasionally with a palpable mass or pathological fracture [2,7]. In our case, the patient had no pain prior to the pathological fracture. Initial evaluation by plain radiograph can show an osteolytic and expansile lesion, usually located in the metaphysis [7] and possibly with a sclerotic margin [2]. Magnetic resonance imaging can support the diagnosis showing characteristic fluid–fluid levels caused by blood sedimentation [22]. The current case showed characteristic lesions in the plain radiograph as well as on MRI. Confirmation of the diagnosis by biopsy is essential, which was applicable in our case. Histopathological findings showed multi-septic cavities filled with blood, the septa containing spindle cells, osteoclast-like giant cells, and inflammatory cells [3,22]. Taking into consideration the results of the imaging studies and histopathological findings, an ABC had to be considered despite the advanced age of the patient.

There is no consensus regarding the optimal treatment of ABCs [18]. In the current case, we decided to treat the patient by complete resection and subsequent reconstruction. En bloc resection has shown excellent results in terms of local control, with a recurrence rate as good as 0% in other studies [18,23,24]. Local treatment such as curettage was not possible in our case due to the pathological fracture already present. In other studies, resection showed a low recurrence rate in patients with ABCs localized in the hand [23,24]. Jafari et al. reported one case with a local recurrence in a patient with an ABC in a metacarpal bone [25]. We discussed with the patient the option of resection and reconstruction using a 3D-printed, patient-specific scaffold made of polylactide and β-tricalcium phosphate and then colonization with autologous stem cells from abdominal fat. The advantage of this method would be a lack of donor site morbidity. However, the patient decided against this because it was too experimental for her and it lacked in vivo results [26]. To date, 32 months postoperatively, we could not detect a recurrence in our case.

Reconstruction of the distal radius can be challenging due to the high functional demands with the goal of preserving the joint function and thus, the range of motion and strength; it can be achieved with a fibula graft. Reconstruction of the distal radius after a secondary ABC has been described previously [27]. However, we highlighted the use of computer-assisted planning and 3D-printed, patient-specific osteotomy templates which improved the efficiency and precision of the surgery. It also facilitated the reconstruction of the physiological shape of the distal radius metaphysis. We recommend this approach in similar cases whenever technical resources are available. However, our case has shown that vascular anatomy should be considered in order to preserve the pedicle.

Our approach achieved excellent results in terms of radiographic consolidation and range of motion. Compared to other studies, our patient achieved a higher range of motion after the reconstruction with a vascularized fibula graft [28,29,30]. Moreover, our patient has not experienced any restrictions in everyday life.

## 5. Conclusions

ABCs are rare, thus establishing a correct diagnosis is essential. In the case of a secondary ABC, tumor formation can be an underlying cause. For this reason, a correct differentiation should be made between primary and secondary ABCs. In our case, an atypical constellation was found consisting of the advanced age of the patient and the presentation of the ABC secondary to a distal radius fracture.

Patient-specific 3D planning is a promising approach to planning functional restoration. As demonstrated in our example, vascular anatomy is critical. The impact of donor-site vascular variability on the surgical strategy should be the subject of further research. Overall, it is presented as a valuable option for the complex reconstruction of the distal forearm.

## Figures and Tables

**Table 1:** The range of motion of the patient after 6 and 18 months after reconstruction and 6 weeks after plate removal (21 months after reconstruction).

	6 Months Postoperatively	18 Months Postoperatively	21 Months Postoperatively, 6 Weeks After Plate Removal
Pro-/Supination [°]	85/0/90	90/0/90	80/0/80
Left	90/0/90		
Right			
Flexion/Extension [°]			
Left	65/0/50	70/0/80	60/0/50
Right	75/0/60		
Radial-/Ulnar deviation [°]			
Left	20/0/20	20/0/40	20/0/40
Right	20/0/40		
Grip-Strength Jamar [kg]			
Left	18		
Right	D30		

## Data Availability

The data presented in this study are available on request from the corresponding author.
